# First record of rainbow shrimp, exotic species *Mierspenaeopsis sculptilis* (Heller, 1862), in the Brazilian coastal amazon, validated by DNA barcode

**DOI:** 10.1186/s40850-023-00176-7

**Published:** 2023-08-11

**Authors:** Charles Samuel Moraes Ferreira, David Carvalho de Mesquita, Ítalo Antônio de Freitas Lutz, Ivana Barbosa Veneza, Thaís Sousa Martins, Paula da Conceição Praxedes Santana, Josy Alessandra Barreto Miranda, Jefferson Miranda de Sousa, Suane Cristina do Nascimento Matos, Francisco Carlos Alberto Fonteles Holanda, Maria Iracilda da Cunha Sampaio, Grazielle Fernanda Evangelista-Gomes

**Affiliations:** 1 Laboratory of Applied Genetics, Institute of Coastal Studies, Federal University of Pará, Bragança, PA Brazil; 2 Laboratory of Fisheries and Navigation, Institute of Coastal Studies, Federal University of Pará, Bragança, PA Brazil; 3 Federal University of Western Pará, Monte Alegre, PA Brazil; 4 Laboratory of Genetics and Molecular Biology, Institute of Coastal Studies, Federal University of Pará, Bragança, PA Brazil

**Keywords:** Crustaceans, Blue Amazon, Conservation, Exotic species

## Abstract

**Background:**

This is the first record of the alien shrimp *Mierspenaeopsis sculptilis* in Brazil. The invasion was detected within Marine Extractive Reserves based on eight specimens accidentally caught by local fishermen using trawlnets focused on fisheries of native species. These specimens were transported to the Laboratory of Applied Genetics and morphologically identified as *Mierspenaeopsis sculptilis* (rainbow shrimp). The taxonomic status of analyzed samples was confirmed by DNA barcoding using a 627-bp fragment of the Cytochrome C Oxidase Subunit I (COI) gene.

**Results:**

A single haplotype was recovered from the eight specimens, being identical to a haplotype reported in India, where this species naturally occurs, and in Mozambique, where the rainbow shrimp is considered an invasive species. The present analyses indicated a putative invasive route (i.e., India-Mozambique-Brazil) mediated by shipping trade.

**Conclusions:**

This study presents the first record of *Mierspenaeopsis sculptilis* in Brazil, in areas of extractive reserves on the Amazon coast. Notably exotic species can cause imbalance in the ecosystem, harming native species. In view of this, the registration of new invasions is essential as they contribute to the implementation of control plans.

## Background

The species *Mierspenaeopsis sculptilis* [1], popularly known as rainbow shrimp, belongs to the Penaeidae family and is originally distributed throughout the western Indo-Pacific region, including northeastern Australia, north of the Bay of Bengal, west coast of India and southeastern Africa [2]. Its diet consists mostly of molluscs and other crustaceans [3].

This shrimp is an important extractive fisheries resource in the Indo-West Pacific region [2]. However, in other places, as invasive species, they can bring numerous risks to the local environment, by threatening local species [4]. They can harm the balance of the local ecosystem in different ways [4], as precursors of diseases and causing changes in the food webs of native species [5]. Therefore, biological invasions are among the most important environmental issues across the globe [6], and are viewed with great concern, especially when they occur in biodiversity hot spot areas [7].

In this scenario, the Amazon, which has an estimated extension of 8.12 million km2 [8], is home to a great diversity of species, which may be threatened by the establishment of exotic species [9], considering that biological invasions have caused major negative socioeconomic and ecological impacts worldwide [10, 11]. Therefore, new records of biological invasions are of great importance, so that they can serve as a warning to the competent environmental authorities and subsidize the adoption of measures to control the spread of exotic species and to protect native species.

Parallel to this, molecular approach has strongly been used worldwide by contributing to the unequivocal identification of species, with emphasis on the portion of DNA known as barcode, a fragment of approximately 650pb of the mitochondrial Cytochrome Oxidase C Subunit I (COI) gene, through which it has been possible to accurately discriminate crustacean species [12–15] and helping for the validation of invasion records.

In addition to recording, it is important to reconstruct possible invasion routes, based on the geographic distribution of identified haplotypes, using mitochondrial DNA from native and invasive populations [16]. Thus, this research identified for the first-time specimens of *M. sculptilis*, based on morphology and the DNA barcoding tool, validating the existence of this shrimp in Brazil, the possible origin and further suggesting the dispersal vector, specifically in different Extractive Reserves of coastal Amazon regions.

## Results

### Characterization of samples and morphological and molecular identification

Among the specimens collected at RESEX Araí-peroba, (Esp_invasor06, Esp_invasor07 and Esp_invasor08), two were females and one male. Both specimens collected at RESEX Caeté-Taperaçú and RESEX Gurupi-Piriá, (Esp_invasor05 and Esp_invasor04), were males. For the specimens: Esp_inavor01, Esp_invasor02 and Esp_inavsor03, sexing identification was not performed since they were without the pleopods. The proportion of sexed individuals was 33.3% female and 66.7% male. The average weight of females (5.82 g) was lower than the average weight of males (12.93 g). Esp_Invasor08 had no face, which made it impossible to obtain some measurements (Table 1). None of the females was ovigerous.

**Fig. 1:**
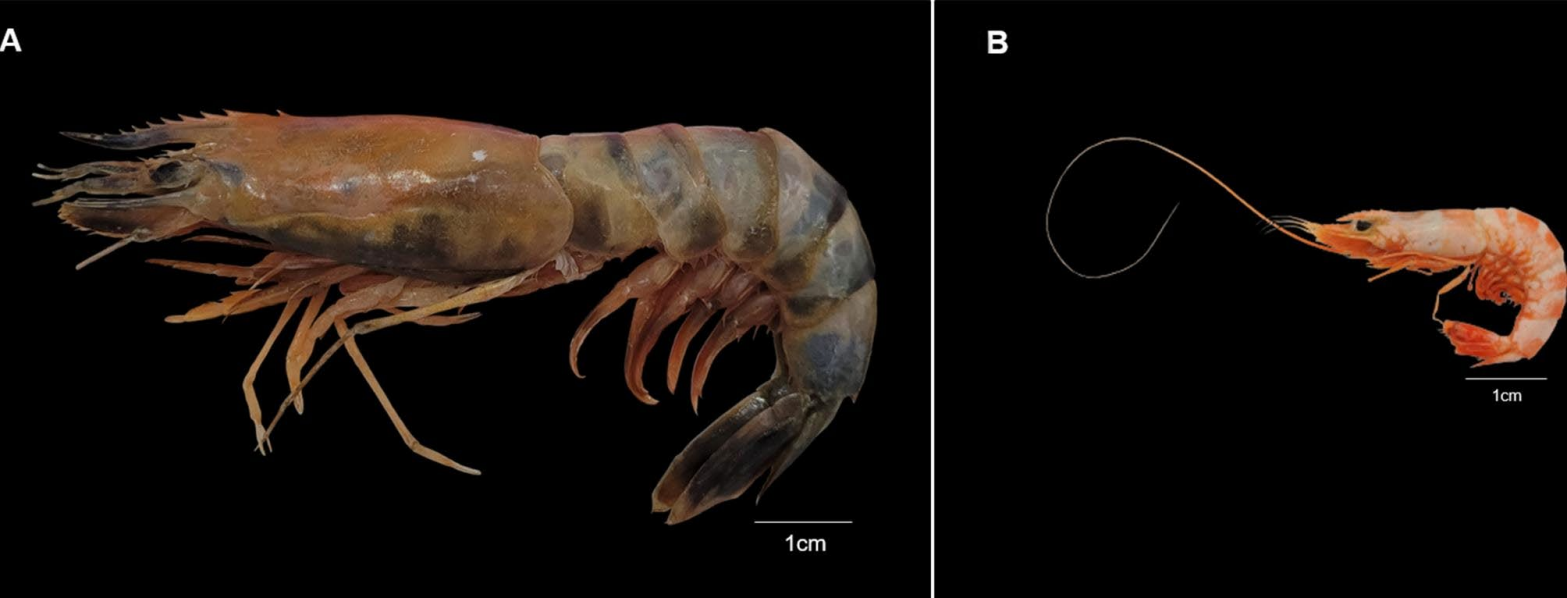
Specimens of *Mierspenaeopsis sculptilis* (**A**) Esp_invasor05, fresh specimen collected in Inferninho beach– RESEX of Caeté-Taperaçú, Bragança; (**B**) Esp_invasor01, salted specimen commercialized in the street market of Bragança

All specimens, except the cooked specimens, could not be analysed due to the cooking and salting processes, and they were identified to the species level as *M. sculptilis*. In (Fig. 1), we present a fresh and a salted specimen.

**Table 1:** Biometric analyses of collected shrimps from the present study

Code	Weight	CT	CR	CFT	AB	Télson	Length CFT	Height CFT	Sex
Esp_Invasor04	8.57	10.1	2.7	5.2	4.7	1.4	1.4	1.5	Female
Esp_Invasor05	13.85	12.5	3.1	6.2	6.3	1.6	1.5	1.8	Male
Esp_Invasor06	4.17	9.8	2.6	4.7	5.1	1.1	1.1	1.1	Female
Esp_Invasor07	4.73	10.2	2.5	4.8	5.4	1.3	1.1	1.3	Female
Esp_Invasor08	12.02	-	-	-	6.9	1.6	1.4	1.7	Male

The weight is shown in grams and the length in centimeters. TL: total length; RL: rostrum length; CFT: cephalothorax length; AB: abdomen length; CFTw: cephalothorax width; CFTh: cephalothorax height

**Fig. 2:**
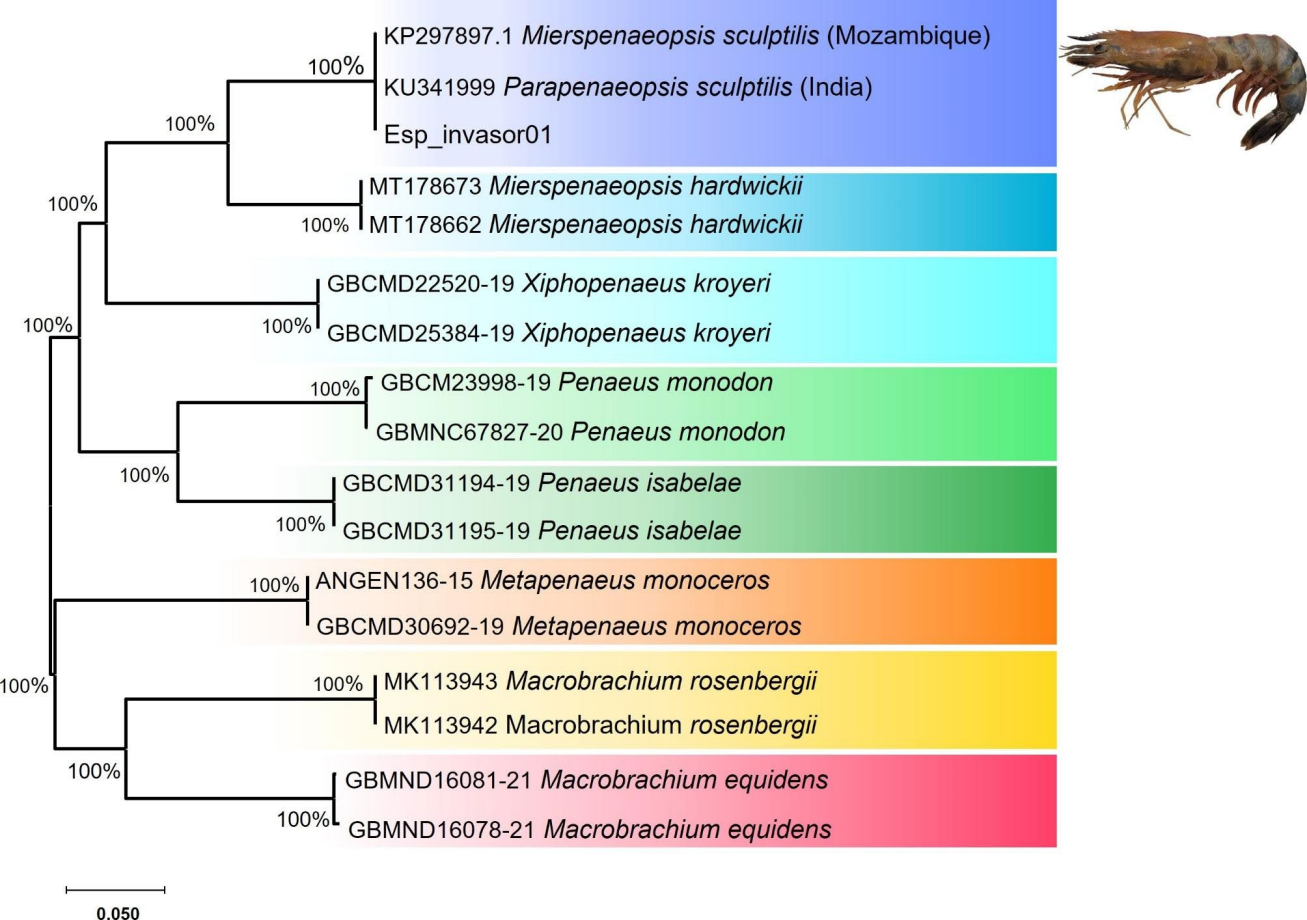
NJ tree. Neighbor-joining phylogenetic tree based on COI haplotypes of *Mierspenaeopsis sculptilis* collected along the Brazilian Amazon coast and reference sequences from public databases. The percentage values refer to the bootstrap estimates

All eight individual sequenced in the present study were recovered as a single haplotype and the result of its submission to the GenBank and BOLD Systems databases, confirmed the identification as *M. sculptilis*, with a similarity percentage of 100% in GenBank, with a haplotype from Mozambique, code KP297897 and 99.81% in BOLD Systems, with a haplotype registered in India, code ANGEN100-15 (Table 2). The distance matrix between and within the analysed groups are in the (Table 3). This result was supported by the topology of the phylogenetic tree, which accurately discriminated the invasive species, recovering a consensus group, reciprocally monophyletic, gathering *M. sculptilis* individuals deposited in public banks and one of the sequences representing the unique haplotype recovered from our samples, with high support value (100% bootstrap) (Fig. 2).

**Table 2:** Taxonomic and molecular identification of *Mierspenaeopsis sculptilis* collected in the present study

Morphological identification			Molecular identification			
Code	Hap	Morphology	GenBank		BOLD *System*	
			Similarity (%)	Acess nº	Similarity (%)	Acess nº
Esp_invasor01	Hap 1	*-*	*M. sculptilis* (100)	KP297897	*M. sculptilis* (99.81)	ANGEN100-15
Esp_invasor02	Hap 1	*-*	*M. sculptilis* (100)	KP297897	*M. sculptilis* (99.81)	ANGEN100-15
Esp_invasor03	Hap 1	*M. sculptilis*	*M. sculptilis* (100)	KP297897	*M. sculptilis* (99.81)	ANGEN100-15
Esp_invasor04	Hap 1	*M. sculptilis*	*M. sculptilis* (100)	KP297897	*M. sculptilis* (99.81)	ANGEN100-15
Esp_invasor05	Hap 1	*M. sculptilis*	*M. sculptilis* (100)	KP297897	*M. sculptilis* (99.81)	ANGEN100-15
Esp_invasor06	Hap 1	*M. sculptilis*	*M. sculptilis* (100)	KP297897	*M. sculptilis* (99.81)	ANGEN100-15
Esp_invasor07	Hap 1	*M. sculptilis*	*M. sculptilis* (100)	KP297897	*M. sculptilis* (99.81)	ANGEN100-15
Esp_invasor08	Hap 1	*M. sculptilis*	*M. sculptilis* (100)	KP297897	*M. sculptilis* (99.81)	ANGEN100-15

The similarity estimates in molecular data were obtained by comparisons with COI sequences available in both GenBank and BOLD (The Barcode of Life Data) Systems platforms

**Table 3:** K2P-based mean genetic distance found within and between the *Mierspenaeopsis sculptilis* specimens from Brazil, India and Mozambique studied here

Within group mean distance		Between group mean distance			
Country	Mean distance (%)	Group	Brasil	India	Mozambique
Brazil	0,00%	Brazil			
India	0,00%	India	0,00%		
Mozambique	0,20%	Mozambique	0,20%	0,20%	

### Scatter vector

**Fig. 3:**
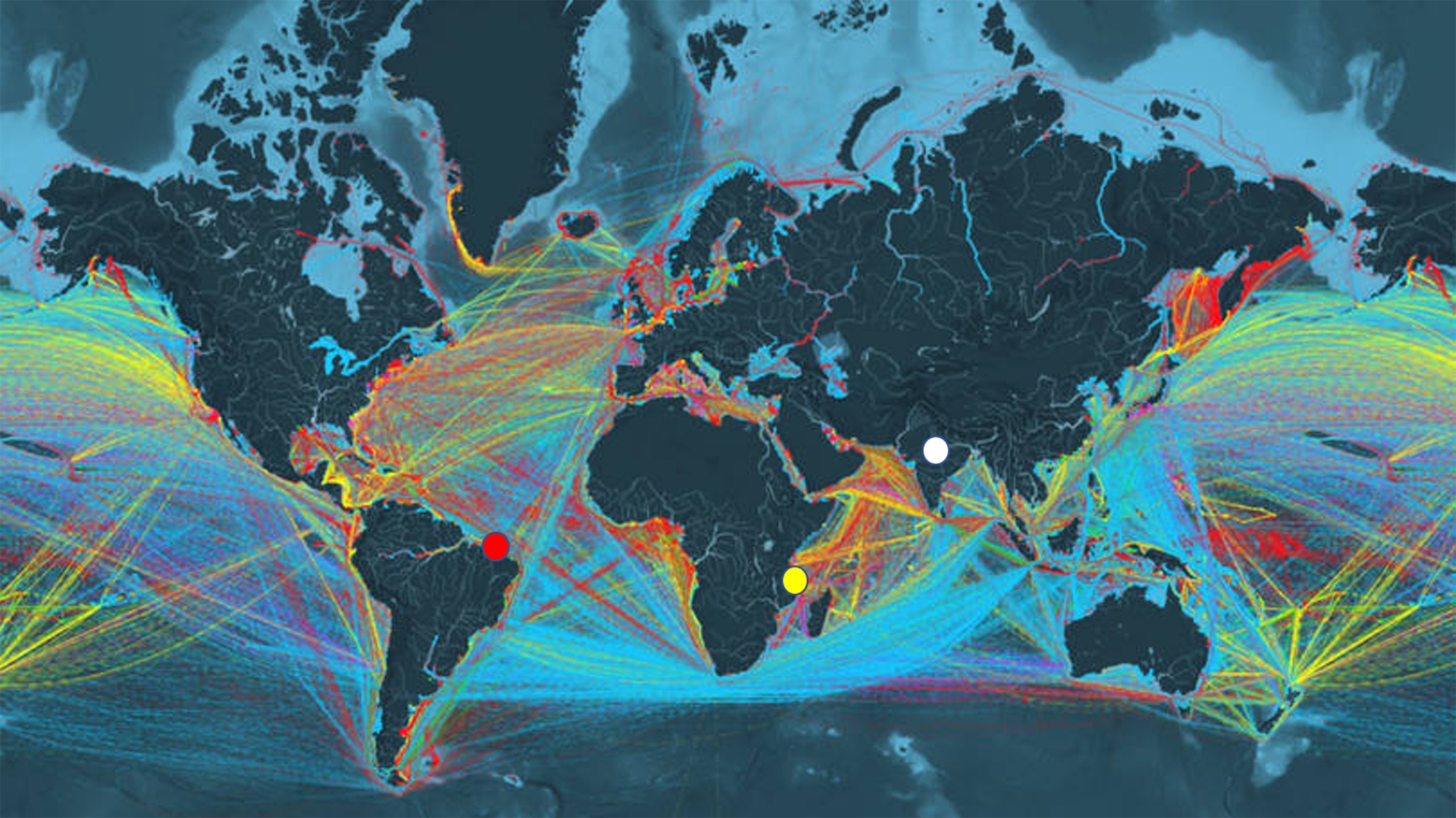
Cargo ship routes. Cargo ship routes developed by the Institute of Energy at University College London, UK and designed by Kiln Digital. Data are based on ships that circulated in 2012. Colored lines: ship routes; red circle: collection sites of rainbow shrimp specimens in Brazil; yellow circle: Mozambique; white circle: India (image downloaded and adapted from https://www.shipmap.org/)

India and Brazil are trading partners, which makes the flow of cargo ships intense, and Mozambique is in the middle of this route (Fig. 3), which leads us to infer that cargo ships are the vectors of this dispersion.

## Discussion

### Unequivocal identification

Based on morphological identification, and molecular analysis, we provide the first record of the occurrence of the invasive rainbow shrimp *Mierspenaeopsis sculptilis* in Brazillian waters based on the DNA barcoding approach. The DNA Barcoding tool have been largely used to efficiently identify shrimp [14, 17, 18].

The genetic distance matrix recovered a high level of similarity within and between specimens from Brazil, Mozambique, and India. The highest genetic distance within the groups was found to be from Mozambique specimens (0.20%), while the samples from Brazil and India showed 0.00% of genetic distance. Regarding the distances between the groups, the samples from Brazil and India do not show divergence (0.00%), while the samples from Mozambique differ by 0.20% from the group from Brazil and India. The similarity between the populations contributes to highlight the maternal origin of the specimens collected in our study.

In the phylogenetic tree, the individual identified as *Parapenaeopsis sculptilis* was deposited before the proposition of the new genus *Mierspenaeopsis* [19]. In addition, the topology of the phylogenetic tree accurately discriminated groups of species that occur in the same natural region of *M. sculptilis*, within the Indo-Pacific region, (*Mierspenaeopsis hardwickii, Metapenaeus monoceros*, *Macrobrachium rosembergii, Penaeus monodon and Macrobrachium equidens*), in addition to accurately discriminating species that naturally occur in Brazil, in the areas where invasive species are captured (*Xiphopenaeus kroyeri* and *Penaeus isabelae*) (Fig. 2).

Among the collection sites, there are points in natural environments, where native shrimp specimens are collected, and at a fish trade fair, located in the municipality of Bragança, state of Pará, which raises the question of a possible establishment between assemblages of native species and a larger scale commercialization of shrimp, already morphologically mischaracterized, at the municipal fair. However, due to the small capture of individuals and because this is the first record of occurrence of this species in Brazilian waters, this issue needs further research.

### Scatter vector

We believe that the vectors of this dispersion are cargo ships, as has already been reported for the invasion of various Decapod crustaceans [20–22], since the rainbow shrimp is not included among the national aquaculture target species and following this, Brazil has an established and active commercial relationship with countries in the Indo-Pacific region [23]. Such commercial relations make the flow of ships intense. Despite this, the Brazilian coast has several commercial ports, which can be considered as focal points for the introduction of marine species [24–26], both due to ballast water and biofouling from ships [27]. Ballast water has been used on ships for more than 50 years to control falls, drafts and stability and has provided an important contribution as a global dispersal vector for aquatic invasive species according to a robust 30-year data assessment [28].

Another important data supporting this hypothesis is that the haplotype recovered from individuals from Brazil is the same haplotype recovered from Mozambique, where this species is also invasive [29]. This haplotype is still present in India, one of the places of natural occurrence of the species [2]. Mozambique is on the route of ships traveling from the Indo-Pacific region to Brazil and from Brazil to the Indo-Pacific region (Fig. 3). Possibly, this dispersal event followed the India ◊Mozambique ◊ Brazil flow. There are numerous biological invasions on the Brazilian coast with the presence of shrimp species of Indo-Pacific origin: *Penaeus monodon* [30]; *Macrobrachium equidens* [31]; *Macrobrachium rosenbergii* [32] and fish: *Butis koilomatodon* [33]; *Pterois volitans* [34] and *Helostoma temminckii* [35].

### Threat to local biodiversity and sustainability of traditional communities

The rainbow shrimp is a species that generally adopt carnivorous diet, consuming mainly molluscs and other crustaceans [3], increasing the threat to shrimp species native to Brazil, which, in addition to competition for resources, are still at risk of being predated by the invasive species [36]. Furthermore, this is scenario may cause the spreading of diseases, which can put the local fauna at risk [37], and may compromise the livelihood of communities, as this biological invasion is taking place in Extractive Reserve areas (RESEX), which are areas used by traditional populations, whose subsistence is based on the withdrawal of natural resources from these areas [38].

## Final considerations

This research presents the first record of *Mierspenaeopsis sculptilis* in Brazil down to the Amazon, in extractive reserves. These results are important as they can help in the management and monitoring strategies of these ecosystems, and to protect local biodiversity, preserving native species and thus maintaining the livelihoods of traditional populations that depend on the native shrimp.

We raise the possibility that exotic shrimp are established in local ecosystems; therefore, we encourage further research to analyse this hypothesis. Finally, we infer that the dispersion vectors are the cargo ships that travel from the Indo-Pacific route, passing through Mozambique and arriving in Brazil.

## Materials and methods

### Sampling

**Fig. 4:**
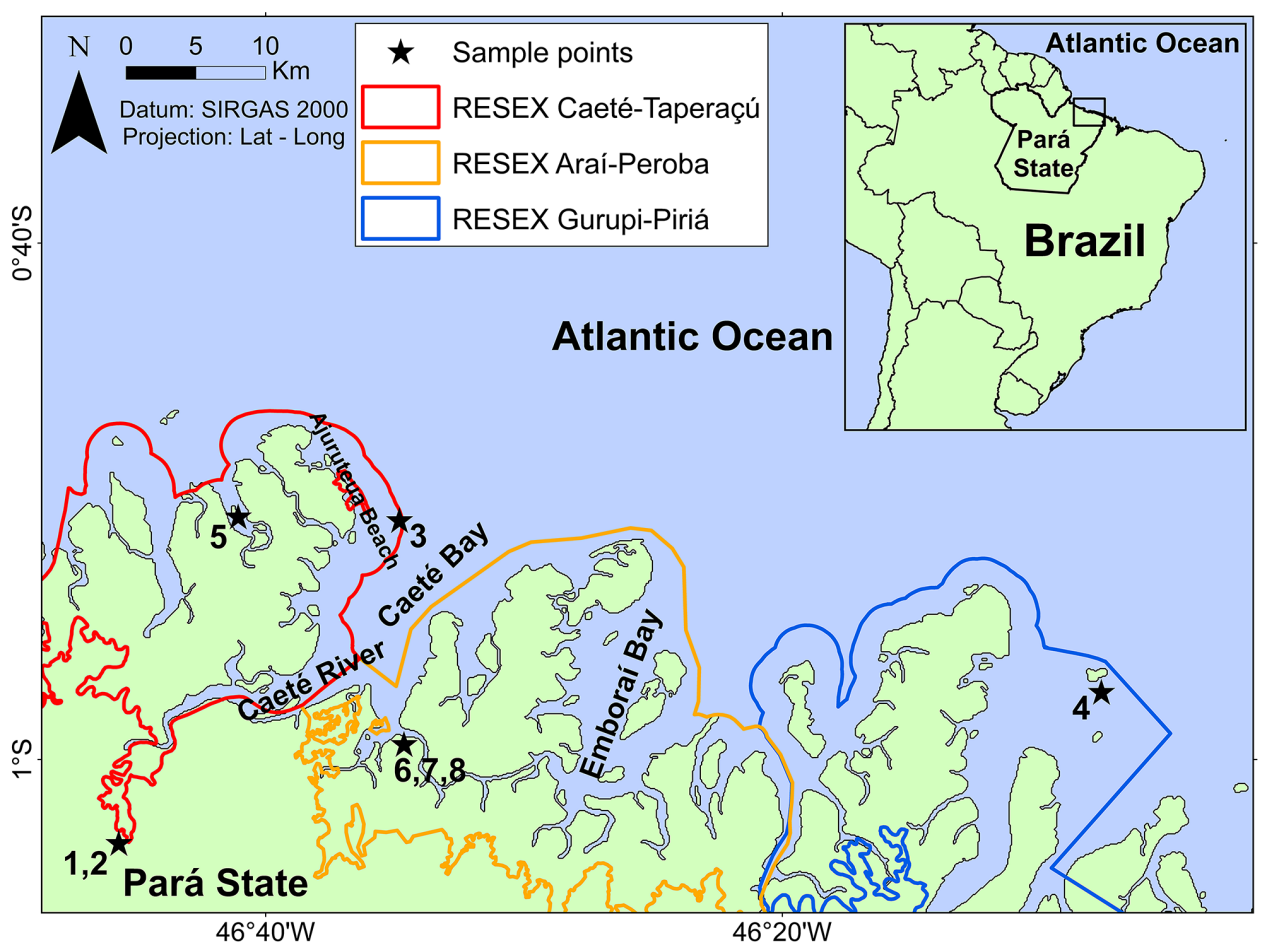
Map of Brazilian Amazon coast showing the collection sites of rainbow shrimp *Mierspenaeopsis sculptilis*. The number refer to the specimen’s code per locality, as follows: 1,2: Street market, Bragança; 3: Fisherman village, Bragança; 4: Lombo Branco Island, Viseu; 5: Inferninho beach, Bragança; 6, 7, 8: Araí, Augusto Corrêa

**Fig. 5:**
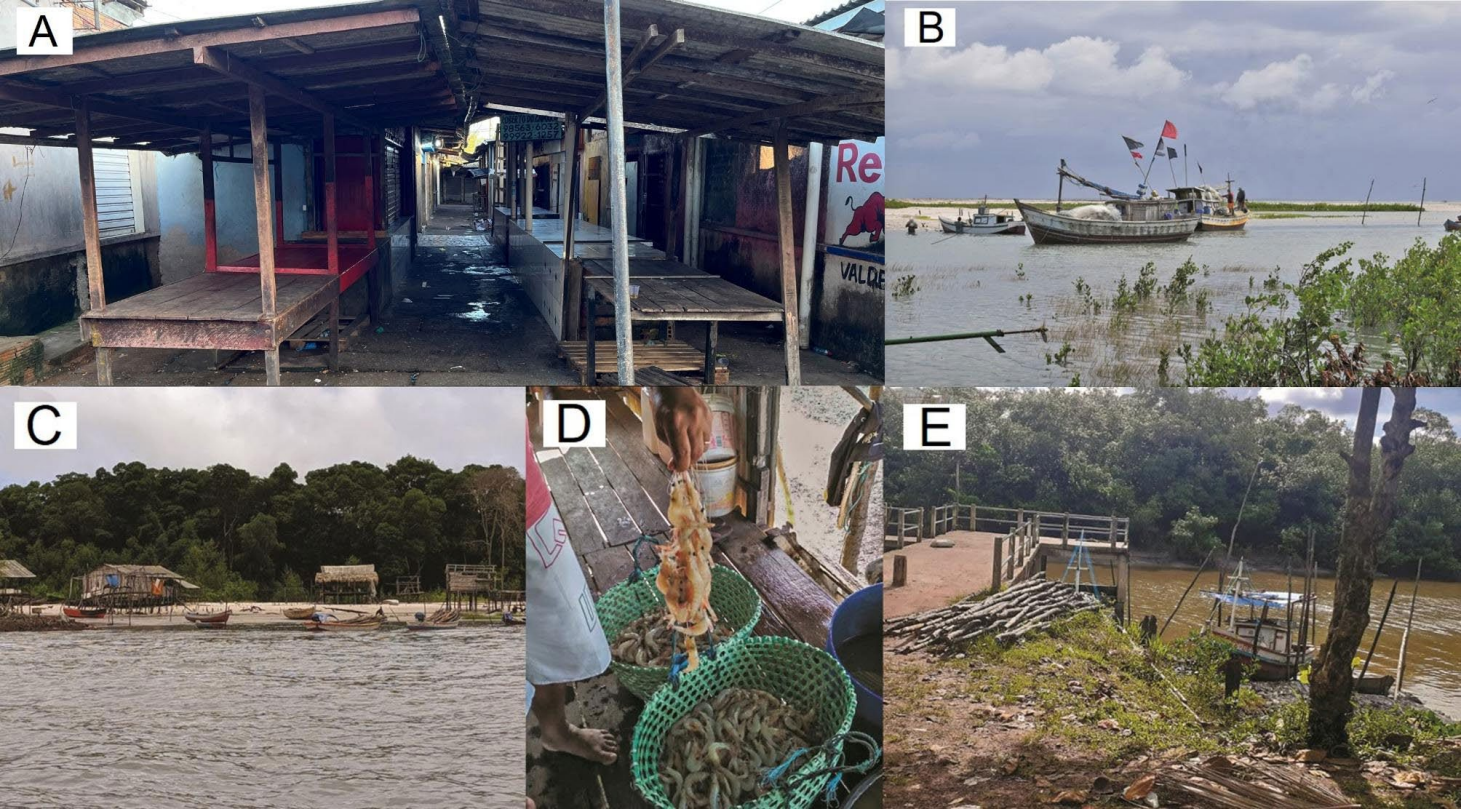
Representative images of collection sites of *Mierspenaeopsis sculptilis* specimens along the Brazilian Amazon coast. (**A**) Street market of Bragança; (**B**) Fisherman Village in Bragança; (**C**) Inferninho beach, RESEX Caeté-Taperaçú; (**D**) Lombo Branco Island, RESEX Gurupi-Piriá; (**E**) Araí Port, RESEX Araí-Peroba

Eight specimens of *M. sculptilis* were captured accidentally by local artisanal fishermen that direct their fishing to a native shrimp species, with trawl nets (20 mm), in the estuary of the Caeté River. Two specimens were in the Extractive Reserve (RESEX) Caeté-Taperaçú; one in the RESEX Gurupi-Piriá and three specimens were located in Araí-peroba in addition to two collected specimens being marketed at a municipal fish fair, located in the municipality of Bragança, coastal Amazon. These last two were bought salted, among other shrimps, under the commercial designation of “shrimp grazado”. The collection sites are in the State of Pará, North of Brazil, and the georeferenced points are detailed in (Table 4). The spatial arrangement of the collected individuals can be seen in (Fig. 4) and images of the places where the specimens were captured are shown in (Fig. 5).

**Table 4:** Collection sites of *Mierspenaeopsis sculptilis* specimens in the Brazilian coastal Amazon, state of Pará

Código	Nome da localidade	Local da coleta		RESEX
		Latitude	Longitude	
Esp_Invasor01^a^	Feira Municipal, Bragança-PA	1º03’13.83”	46º45’40.93”	-
Esp_Invasor02^a^	Feira Municipal, Bragança-PA	1º03’13.83”	46º45’40.93”	-
Esp_Invasor03	Vila dos Pescadores, Bragança-PA	0º50’44.62”	46º34’24.81”	Caeté-Taperaçú
Esp_Invasor04	Ilha do Lombo Branco, Viseu-PA	0°57’22.61”	46°07’38.72”	Gurupi-Piriá
Esp_Invasor05	Praia do Inferninho, Bragança-PA	0º50’35.05”	46º41’03.69”	Caeté-Taperaçú
Esp_Invasor06	Porto do Araí, Augusto Corrêa-PA	0°59’47.73”	46°20’12.99”	Araí-Peroba
Esp_Invasor07	Porto do Araí, Augusto Corrêa-PA	0°59’47.73”	46°20’12.99”	Araí-Peroba
Esp_Invasor08	Porto do Araí, Augusto Corrêa-PA	0°59’47.73”	46°20’12.99”	Araí-Peroba

^a^Market salt sample

RESEX: Extractive Reserve

The samples were taken to the Laboratory of Applied Genetics (LAGA), of the Institute of Coastal Studies (IECOS), Federal University of Pará (UFPA), in Bragança, where a small fragment (2 cm) of muscle tissue was removed from everyone, for individual storage in 2mL eppendorf microtubes, containing 90% alcohol, and conditioned in a freezer at a constant temperature of -20 °C. All, except for the salted individuals, were fixed in 10% formaldehyde and preserved in 70% alcohol, to compose the laboratory’s Zoological Collection, as testimonial specimens. Salted samples were immediately processed upon arrival at the laboratory. All specimens used in this research came from an artisanal fishery and from municipal fair. No live specimen was manipulated. Therefore, no ethical approval was necessary.

### Morphological identification

All specimens, except for salted samples, underwent biometrics, in which the weight of fresh specimens was recorded using digital scales (precision of 0.1 g), the total length (CT), which was defined as the distance from the tip of the rostrum to the distal end of the telson. Lengths were measured using a pachymeter (accuracy of 0.01 mm). The sex of each individual was identified by the presence or absence of the male appendix on the second pair of pleopods and they were morphologically identified to the species level, using an identification key [19].

### Molecular identification: obtaining of genetic marker and DNA sequencing

The total DNA was isolated using the Wizard Genomic DNA (Promega) kit, according to the manufacturer’s instructions. The quality of DNA samples was evaluated by electrophoresis in 1% agarose gel stained with BlueJuice™ Gel Loading Buffer (Ludwingbiotec) and GelRed® Nucleic Acid Stain (Ludwingbiotec).

The barcode fragment of COI gene was amplified through the Polymerase Chain Reaction – PCR approach, using primers LCO-1490 and HCO-2198 [39]. Positive PCR products were purified with PEG 8000 (Polyethylene Glycol), according to [40], and subsequently sequenced using the dideoxyterminal method [41]. with reagents from the Big Dye Kit 3.1 (ABI Prism TM Dye Terminator Cycle Sequencing Ready Reaction — PE Thermo Fisher), following the manufacturer’s recommendations. After the sequencing reaction, the precipitated product was subjected to electrophoresis in the ABI™ 3500 XL automatic capillary sequencer (Thermo Fisher).

### Database and genetic analysis

Eight COI sequences were generated, all of them underwent electropherogram inspection individually, in the BioEdit v. 7.2.5 [42], for evaluation and correction of possible errors. The automatical alignment was done through ClustalW tool [43, 44], implemented in BioEdit v. 7.2.5 [42]. After inspection and alignment, a consensus bank with 627 base pairs was obtained. Then, the database was analyzed in DNAsp v6 [45] for the identification of haplotypes, to guide the identification process.

The identified haplotype was submitted to the public plataform GenBank (National Center for Biotechnology Information - http://www.ncbi.nlm.nih.gov) and to the BOLD Platform (Barcoding of Life Database - http://www.barcodinglife.org) [46], for the process of molecular identification.

To assemble the final database, three sequences were obtained from GenBank, two of *M. sculptilis* and one as an outgroup including other shrimp species, constituting a final database with seventeen sequences with consensus length of 508 base pairs. This database was used to build a Neighbor-Joining tree (NJ), using the (K2P) model proposed by Kimura, [47], with support values estimated by the Booststrap method [48], based on 1000 pseudoreplicates, in the Mega X program [49]. The K2P model was also used to generate an intra and interspecific distance matrix between specimens from Brazil, Mozambique and India in the aforementioned program.

## Data Availability

The datasets generated during and/or analyzed during the current study are available from the corresponding author on reasonable request.

## Abbreviations

ABI: Applied Biosystems™
AB: Abdomen length
BOLD: Barcoding of Life Database
COI: Cytochrome Oxidase C Subunit I
CFT: Cephalothorax length
CFTw: Cephalothorax width
CFTh: Cephalothorax height
CT: Total length
DNA: Deoxyribonucleic Acid
°C: Degrees Celsius
IECOS: Institute of Coastal Studies
K2P: Kimura two-parameter
LAGA: Laboratory of Applied Genetics
NJ: Neighbord-Joining
PCR: Polymerase Chain Reaction
PEG: Polyethylene Glycol
RESEX: Extractive Reserve
RL: Rostrum length
SECEX: Secretary of Foreign Trade
TL: Total length
UFPA: Federal University of Pará
